# Emergence of multidrug-resistant non-fermentative gram negative bacterial infection in hospitalized patients in a tertiary care center of Nepal

**DOI:** 10.1186/s13104-020-05163-6

**Published:** 2020-07-02

**Authors:** Santosh Kumar Yadav, Rajshree Bhujel, Shyam Kumar Mishra, Sangita Sharma, Jeevan Bahadur Sherchand

**Affiliations:** 1 Department of Microbiology, Rajarshi Janak University, Janakpurdham, Nepal; 2 Department of Clinical Microbiology, Institute of Medicine, Tribhuvan University Teaching Hospital, Kathmandu, Nepal

**Keywords:** *Acinetobacter baumannii*, Hospitalized patients, Multidrug-resistant, Non-fermentative gram negative bacteria, *Pseudomonas aeruginosa*

## Abstract

**Objective:**

This study was designed for the characterization and establishment of antibiotic susceptibility profiles of non-fermentative gram negative bacteria isolated from hospitalized patients in a tertiary care hospital of Nepal.

**Results:**

A total of 402 non-fermentative gram negative bacteria was isolated in 1486 culture-positive cases from 6216 different clinical samples obtained from hospitalized patients. Among total non-fermentative gram negative bacterial isolates, the highest number was recovered from specimens collected from lower respiratory tract infections (n = 173, 43.0%) of hospitalized patients followed by pus/swab samples (n = 99, 24.6%) and urinary tract infections (n = 49, 12.2%). The most common non-fermentative gram negative bacteria identified were *Acinetobacter baumannii* (n = 177, 44.0%), *Pseudomonas aeruginosa* (n = 161, 40.1%) and *Burkholderia cepacia* complex (n = 33, 8.2%). These bacterial isolates exhibited a higher rate of insusceptibility to beta-lactam antibiotics, fluoroquinolones, and aminoglycosides. On the other hand, all the isolates of *P. aeruginosa* and *A. baumannii* were completely susceptible to colistin sulfate and polymyxin B. Among total isolates, 78.1% (n = 314) were multidrug-resistant with a high rate of multidrug-resistant among *A. baumannii* (91.0%).

## Introduction

Non-fermentative gram negative bacteria (NFGNB) are a heterogeneous group of bacteria that includes *Pseudomonas* species*, Acinetobacter* species*, Burkholderia* species*, Stenotrophomonas maltophilia,* etc. These organisms are omnipresent and may be recovered from various hospital instruments as well as from the body surface of healthcare workers [1]. They can cause a vast variety of infections in hospitalized patients and account for approximately 15% of all gram negative bacterial infections [2]. NFGNB pose significant challenges in health care settings because of their multiple, intrinsic, or acquired antibiotic resistance. The burden of resistance is presumably more due to the higher rate of empirical antimicrobial treatment than with the virulence of particular strains [3]. NFGNB are becoming a threat to health care systems because these bacteria are mainly associated with opportunistic infections in critically ill and immunocompromised patients. The emergence of infections by these organisms along with the rising drug resistance among them warrants close monitoring of the antimicrobial susceptibility profile of these organisms [4]. The objective of this study was to characterize NFGNB and determine its antibiotic susceptibility among hospitalized patients.

## Main text

### Methods

This study was conducted at the department of clinical microbiology of Tribhuvan University Teaching Hospital, Nepal from January 2017 to December 2017. The various specimens collected from hospitalized patients (suspected with infections) were processed for isolation and identification of NFGNB. The lower respiratory tract specimens and body fluid samples were inoculated on chocolate agar (CA) plate, 5% sheep blood agar (BA) plate, and MacConkey agar (MA) plate. Blood samples were initially enriched with Brain Heart Infusion Broth (BHIB) and then subcultured on to the BA and MA plates. Similarly, wound swab, pus, catheter tips, and urine samples were cultured on BA and MA plates. The CA plates were incubated in CO_2_ incubator at 37 °C for 24 h. The BA and MA plates were incubated at 37 °C for 24 h in an aerobic condition [5]. The identification of significant NFGNB isolates that are associated with infections was performed following standard microbiological techniques which involved the morphological appearance of the colonies, Gram’s staining, motility test and a battery of biochemical tests which included oxidase test, sugar fermentation test including triple sugar iron agar, citrate and acetamide utilization test, nitrate reduction test, decarboxylation tests, gelatin liquefaction test, deoxyribonuclease test, etc [6].

The susceptibility of bacterial isolates against different antibiotics was determined by the Kirby–Bauer disk diffusion method and minimum inhibitory concentration (MIC) method for some antibiotics. Interpretations of antibiotic susceptibility test results were made according to the guidelines provided by the Clinical and Laboratory Standards Institute (CLSI) (26th Edition) [7]. *Escherichia coli* ATCC 25922 and *P. aeruginosa* ATCC 27853 were used as the control organisms for the antibiotic sensitivity test. The isolates resistant to at least one antibiotic in three or more antimicrobial classes were confirmed as multidrug-resistant (MDR) phenotype [5, 8].

The data generated during the study were analyzed by using the SPSS version 16.0 and interpreted according to frequency distribution and percentage.

### Results

From a total of 6216 specimens, 1486 (23.9%) showed significant bacterial growth which included 402 NFGNB (27.1% of culture-positive). The isolation rate of NFGNB was highest from lower respiratory tract infection (37.2%) and lowest from urinary tract infection (17.5%). Among the total of 402 NFGNB isolates, 58.5% (n = 235) were isolated from male and 41.5% (n = 167) were from female patients, with male to female ratio 1.41. The highest number of isolates were from the age group 16–32 years (n = 100) and the least number from the age group 49–64 years (n = 66).

From total isolates, nine species of NFGNB were identified which included *Acinetobacter baumannii* (n = 177, 44.0%) followed by *Pseudomonas aeruginosa* (n = 161, 40.1%), *Burkholderia cepacia* complex (n = 33, 8.2%), *A. calcoaceticus* (n = 11, 2.7%), *A. lwoffii* (n = 10, 2.5%), and few other isolates. The majority of *A. baumannii* (n = 82) and *Pseudomonas aeruginosa* (n = 79) were recovered from lower respiratory tract specimens i.e. sputum, bronchoalveolar lavage, and endotracheal aspirates (Table 1). Among wards, 39.1% of total NFGNB were isolated from intensive care unit (ICU) patients, an equal number (21.9%) from each surgical and medical wards followed by orthopedics (6.5%), pediatrics (4.9%), maternity (4.2%) and burn ward (1.5%).

**Table 1:** Distribution of non-fermentative gram negative bacteria in different clinical specimens

NFGNB isolates	Clinical specimens						
	LRTS^a^	Pus/swabs	Urine	Body fluids	Blood	Catheter tips	Total number (%)
*Acinetobacter baumannii*	82	49	13	20	10	3	177 (44.0)
*Acinetobacter calcoaceticus*	2	3	3	2	1	–	11 (2.7)
*Acinetobacter lwoffii*	–	6	2	2	–	–	10 (2.5)
*Acinetobacter haemolyticus*	–	–	3	–	–	–	3 (0.8)
*Pseudomonas aeruginosa*	79	37	21	18	4	2	161 (40.1)
*Pseudomonas stutzeri*	–	1	–	–	1	–	2 (0.5)
*Burkholderia cepacia* complex	8	2	7	5	9	2	33 (8.2)
*Stenotrophomonas maltophilia*	1	1	–	1	1	–	4 (1.0)
*Sphingobacterium species*	1	–	–	–	–	–	1 (0.2)
Total number (%)	173 (43.0)	99 (24.6)	49 (12.2)	48 (12.0)	26 (6.5)	7 (1.7)	402 (100)

^a^ Lower respiratory tract specimens include sputum, bronchoalveolar lavage, and endotracheal aspirate

*Acinetobacter baumannii* and *Pseudomonas aeruginosa* showed a low level of sensitivity to most of the commonly tested antibiotics in clinical practice. When results were critically analyzed for the common organisms amongst these we found that *A. baumannii* and *P. aeruginosa* showed resistance to most of the common drugs except polymyxin B and colistin sulfate. *Acinetobacter calcoaceticus, A. lwoffii, A. haemolyticus,* and *P. stutzeri* showed high sensitivity against the tested antimicrobials. All the isolates of *Burkholderia cepacia* complex and *Stenotrophomonas maltophilia* showed a considerable amount of sensitivity to co-trimoxazole and doxycycline (Table 2). Among total NFGNB isolates, 78.1% were MDR with a high rate among *A. baumannii*. *P. aeruginosa* and *B. cepacia* complex (Table 3).

**Table 2:** Antibiotic susceptibility profile of non-fermentative gram negative bacterial isolates

Antibiotics	Sensitivity rate of non-fermentative gram negative bacteria (%)		
	*Acinetobacter baumannii* (*n* = 177)	*Pseudomonas aeruginosa* (*n* = 161)	*Burkholderia cepacia* complex (*n* = 33)
Ampicillin-sulbactam	18.6	NT	NT
Piperacillin	6.2	21.7	NT
Piperacillin-tazobactam	12.4	38.5	NT
Ceftazidime	6.2	13.0	12.1
Cefotaxime	4.5	NT	NT
Cefepime	6.8	15.5	NT
Meropenem	18.1	38.5	66.7
Imipenem	20.3	42.2	NT
Ciprofloxacin	11.3	12.4	NT
Ofloxacin	NT	13.7	NT
Levofloxacin	19.2	23.0	15.2
Gentamicin	14.1	43.5	NT
Amikacin	20.9	62.7	NT
Co-trimoxazole	4.0	NT	100
Doxycycline	42.9	NT	72.7
Polymyxin B	100	100	NT
Colistin sulfate	100	100	NT
Chloramphenicol	NT	NT	45.5

*NT* antibiotics not tested/not recommended by CLSI

**Table 3:** Multidrug resistance rate in non-fermentative gram negative bacterial isolates

NFGNB isolates (number of isolates)	MDR in NFGNB	
	Number	Percentage
*Acinetobacter baumannii* (n = 177)	161	91.0
*Acinetobacter calcoaceticus* (n = 11)	4	36.4
*Acinetobacter lwoffii* (n = 10)	0	0
*Acinetobacter haemolyticus* (= 3)	0	0
*Pseudomonas aeruginosa* (n = 161)	118	73.3
*Pseudomonas stutzeri* (n = 2)	0	0
*Burkholderia cepacia* complex (n = 33)	26	78.8
*Stenotrophomonas maltophilia* (n = 4)	4	100
*Sphingobacterium* species (*n* = 1)	1	100
Total (N = 402)	314	78.1

### Discussion

Infection in hospitalized patients is a global health problem. Despite awareness and hospital care, infections continue to develop in hospitalized patients [9]. Non-fermentative gram negative bacteria were previously considered less significant pathogens but they have now emerged as important nosocomial pathogens [10]. In our study, a total of 402 NFGNB were isolated from hospitalized patients which are 27.1% of culture-positive samples. A similar growth rate of NFGNB was documented by Bhagava et al. (29.6%) [11] from Nepal and Sharma et al. (25.6%) [9] from India while Rahbar et al. [12] reported a positivity rate of only 15% for NFGNB from Iran. These variations in the prevalence of NFGNB in different health care settings might be due to the infection control practices and circulation of these bacterial pathogens in respective hospitals. The demographic data in our study showed patients most commonly affected by NFGNB infections were male and between age 16–32 years, which is in accordance with the study done by Benachinmardi et al. [13]. In this study, the incidence of infections with NFGNB was found to be highest from lower respiratory tract specimens (43.0%), followed by pus and swabs (24.6%), urine (12.2%), body fluids (12.0%) and blood (6.5%). Nautiyal et al. [14] from India also reported major NFGNB isolates from lower respiratory tract specimens (42.3%) followed by pus samples (28.6%) while Bhatnagar et al. [15] reported most of the NFGNB isolates from pus samples (49.2%) and the next common being sputum sample (19.8%).

Among total isolates, *A. baumannii* was the most common isolates (44.0%) followed by *P. aeruginosa* (40.1%), *B. cepacia* complex (8.2%), and *A. calcoaceticus* (2.7%). Parajuli et al. [5] and Sah et al. [16] also reported *A. baumannii* as the major NFGNB isolates from hospitalized patients. *Acinetobacter baumannii* and *P. aeruginosa* were the most frequently isolated from lower respiratory tract specimens, pus, and urine samples. This result is supported by other studies [10, 17]. *Acinetobacter* species is an important nosocomial pathogen imposing greater problem to the management of hospitalized patients and infection control [18]. Samawi et al. [19] from Qatar also reported major *A. baumannii* from respiratory tract infection. Other *Acinetobacter* species were commonly isolated from pus and swab samples. In this study, *P. aeruginosa* was the second commonest NFGNB accounting for 40.1% of total isolates. Other studies from Nepal [20] and India [9, 21] also revealed a high prevalence of *P. aeruginosa* from respiratory tract infections. Parajuli et al. [5] reported most of the *P. aeruginosa* from VAP, followed by SSI and UTI of ICU patients. In recent years *Burkholderia cepacia* complex has emerged as an important nosocomial pathogen and its multidrug resistance makes its presence dangerous in hospital settings. In our study, most of the *B. cepacia* complex was associated with bloodstream infection (n = 9, 27.3%). Dizbay et al. [22] from Turkey also reported 25.6% *B. cepacia* complex from bloodstream infection. *Stenotrophomonas maltophilia* infections are acquiring increasing importance in the hospital environment. In this study, A total of four *S. maltophilia* were isolated, one from each blood, sputum, body fluid, and pus sample. *Sphingobacterium* species (n = 1) was isolated from the sputum sample of a patient with pneumonia. There are certain reported cases of bacteremia, respiratory tract infection, and meningitis due to *Sphingobacterium* species from various countries [23, 24].

The pattern of NFGNB infections among patients of different wards was evaluated in this study. High prevalence of NFGNB was isolated from patients of intensive care units (39.1%) followed by surgical and medical wards (21.1% each), orthopedic (6.5%), and pediatric (4.9%) wards. Other studies [25, 26] have also reported a higher incidence of NFGNB infection in ICU patients. It is a well-known fact that the infection rates in ICUs are many times higher than elsewhere in hospitals as ICUs are the hub of critically ill patients, who are most vulnerable to opportunistic infections by NFGNB. In our study, the most common NFGNB causing infections in ICU patients were *A. baumannii* (n = 84, 53.5%) followed by *P. aeruginosa* (n = 55, 35.0%) and *B. cepacia* complex (n = 15, 9.6%). *Acinetobacter* spp. as the commonest NFGNB pathogen in ICU patients was also documented by Parajuli et al. [5] and Mishra et al. [27].

This study showed lower susceptibility of different NFGNB against many antimicrobials with a high multidrug resistance rate. As a consequence of easy access and haphazard use, many antibiotics have decreased their effectiveness in Nepal. *Acinetobacter baumannii* isolates showed a very low sensitivity rate (< 25%) against penicillins, cephalosporins, fluoroquinolones, aminoglycosides, and even carbapenems. They were moderately and highly susceptible to doxycycline (42.9%) and polymyxins (100%), respectively. The resistance rate is higher than the previous results from the same hospital [28]. Parajuli et al. [5] and Shrestha et al. [18] reported similar antibiotic susceptibility profiles among *A. baumannii*. Xia et al. [29] documented only 15% *A. baumannii* susceptible to carbapenems. On the other hand, *A. calcoaceticus, A. lwoffii,* and *A. haemolytics* were found highly susceptible to common antimicrobials. *Pseudomonas aeruginosa* isolates were also found to be highly resistant against β-lactams. Among different antibiotics tested, only 13.0% isolates were found to be susceptible to ceftazidime, 15.5% to cefepime, 38.5% to meropenem, 42.2% to imipenem, 12.4% to ciprofloxacin and 23.0% to levofloxacin, while all the isolates were completely susceptible to polymyxin B and colistin sulfate. Fatima et al. [30] and Xie et al. [29] reported a lower resistance rate in *P. aeruginosa* against beta-lactam antibiotics. Our finding well correlates with the observations of other studies [5, 31, 32]. All the isolates of *B. cepacia* complex were found susceptible to co-trimoxazole, 72.7% to doxycycline, 66.7% to meropenem, and only 12.1% to ceftazidime. A similar pattern of susceptibility was reported by Parajuli et al. [5] among ICU patients.

The MDR rate among *A. baumannii* was 91.0% which is extensively high. Shrestha et al. [33] and Mishra et al. [28] also reported around 96% and 95% MDR *A. baumannii,* respectively. We found 73.3% MDR *P. aeruginosa* isolates which are higher than those reported by Mishra et al. [34] from Nepal in 2008 (65.9%), Prakash et al. [35] from India in 2014 (31.7%), and Hassuna et al. [36] from Egypt in 2015 (56%). The rate of MDR *P. aeruginosa* is in an upward trend throughout the world especially in developing countries causing a life-threatening situation. *Stenotrophomonas maltophilia* is intrinsically resistant to many antibiotics and MDR is common. In this study, all the *S. maltophilia* was found to be MDR and only 25.0% isolates were susceptible to levofloxacin and chloramphenicol and 50.0% to doxycycline. Sattler et al. [37] reported a similar resistance rate among *S. maltophilia*.

In this study, polymyxin B and colistin sulfate showed excellent effect against *Acinetobacter* species and *P. aeruginosa* isolates. Co-trimoxazole was found effective against *B. cepacia* complex and *S. maltophilia*. The antibiotic resistance among these bacteria has dramatically increased in the last decades and pose a significant challenge to effective therapeutic strategies. Menacing a state of low antimicrobial susceptibility among NFGNB associated with these infections is particularly worrisome. Therefore, surveillance of antibiotic susceptibility profiles of these bacteria should be regularly done.

## Limitations

We were unable to evaluate the risk factors and outcomes of NFGNB infections in hospitalized patients due to the unavailability of sufficient data. Furthermore, molecular analysis of the resistant phenotypes and genetic mechanism of drug resistance was not determined.

## Supplementary information

**Additional file 1: Table S1.** Distribution of specimens and growth rate of non-fermentative gram negative bacteria. **Table S2.** Distribution of non-fermentative gram negative bacteria with the demographic features of patients. **Table S3.** Ward wise distribution of non-fermentative gram negative bacterial isolates.

## Data Availability

All data generated or analyzed during this study are included in this published article and its additional information files (Additional file 1: Table S1–S3).

## Abbreviations

AST: Antibiotic susceptibility testing
ATCC: American type culture collection
BA: Blood agar
BHIB: Brain Heart Infusion Broth
CA: Chocolate agar
CLSI: Clinical and Laboratory Standards Institute
ICU: Intensive care unit
LRTS: Lower respiratory tract specimens
MA: MacConkey agar
MDR: Multidrug-resistant
MIC: Minimum inhibitory concentration
NFGNB: Non-fermentative gram negative bacteria
SPSS: Statistical Package for Social Sciences
SSI: Surgical-site infection
UTI: Urinary tract infection
VAP: Ventilator-Associated Pneumonia
