# Supplementary material for: Emergence of multidrug-resistant non-fermentative gram negative bacterial infection in hospitalized patients in a tertiary care center of Nepal
Source: BMC Res Notes. 2020 Jul 2;13:319. doi: 10.1186/s13104-020-05163-6 (PMC7330544; doi:10.1186/s13104-020-05163-6)
Supplement: Supplementary file 1 — Additional file 1: Table S1. Distribution of specimens and growth rate of non-fermentative gram negative bacteria. Table S2. Distribution of non-fermentative gram negative bacteria with the demographic features of patients. Table S3. Ward wise distribution of non-fermentative gram negative bacterial isolates. [file 13104_2020_5163_MOESM1_ESM.docx]

**Table S1** Distribution of specimens and growth rate of non-fermentative gram negative bacteria

| Specimen type | Number of specimens | Culture positive | Growth of NFGNB | |
| --- | --- | --- | --- | --- |
|  |  |  | Number | % of culture-positive |
| LRTs^*^ | 996 | 465 | 173 | 37.2 |
| Pus and swabs | 933 | 494 | 99 | 20.0 |
| Urine | 2614 | 280 | 49 | 17.5 |
| Body fluids | 575 | 140 | 48 | 34.3 |
| Blood | 1002 | 87 | 26 | 29.9 |
| Catheter tips | 96 | 20 | 7 | 25.0 |
| Total | 6216 | 1486 | 402 | 27.1 |

^*^ Lower respiratory tract specimens include sputum, bronchoalveolar lavage and endotracheal aspirate

**Table S2** Distribution of non-fermentative gram negative bacteria with the demographic features of patients

| Age Group  (Years) | Number (%) | | Total number (%) |
| --- | --- | --- | --- |
|  | Female | Male |  |
| ≤15 | 36 (46.2) | 42 (53.8) | 78 (19.4) |
| 16-32 | 43 (43.0) | 57 (57.0) | 100 (24.9) |
| 33-48 | 35 (42.7) | 47 (57.3) | 82 (20.4) |
| 49-64 | 29 (43.9) | 37 (56.1) | 66 (16.4) |
| ≥65 | 24 (31.6) | 52 (68.4) | 76 (18.9) |
| Total number (%) | 167 (41.5) | 235 (58.5) | 402 (100) |

**Table S3** Ward wise distribution of non-fermentative gram negative bacterial isolates

| NFGNB isolates | Wards | | | | | | |
| --- | --- | --- | --- | --- | --- | --- | --- |
|  | ICUs | Surgical | Medical | Orthopedic | Pediatric | Maternity | Burn |
| *A. baumannii* (n=177) | 84 | 39 | 26 | 14 | 7 | 4 | 3 |
| *A. calcoaceticus* (n=11) | 1 | 4 | 1 | - | 1 | 4 | - |
| *A. lwoffii* (n=10) | - | 4 | 2 | 2 | - | 2 | - |
| *A. haemolyticus* (n=3) | - | - | - | - | - | 3 | - |
| *P. aeruginosa* (n=161) | 55 | 32 | 51 | 9 | 8 | 3 | 3 |
| *P. stutzeri* (n=2) | - | 1 | 1 | - | - | - | - |
| *B. cepacia* complex (n=33) | 15 | 7 | 6 | 1 | 3 | 1 | - |
| *S. maltophilia* (n=4) | 2 | 1 | 1 | - | - | - | - |
| *Sphingobacterium species* (n=1) | - | - | - | - | 1 | - | - |
| Total number (N=402)  (%) | 157  (39.1) | 88  (21.9) | 88  (21.9) | 26  (6.5%) | 20  (4.9) | 17  (4.2) | 6  (1.5) |
